# Understanding immune signaling using advanced imaging techniques

**DOI:** 10.1042/BST20210479

**Published:** 2022-03-28

**Authors:** Mario Brameshuber, Enrico Klotzsch, Aleks Ponjavic, Erdinc Sezgin

**Affiliations:** 1 Institute of Applied Physics – Biophysics, TU Wien, 1040 Vienna, Austria; 2 Humboldt-Universität zu Berlin, Institut für Biophysik, Experimentelle Biophysik Mechanobiologie, Sitz Invalidenstrasse 42, 10115 Berlin, Germany; 3 School of Physics and Astronomy, University of Leeds, Woodhouse Lane, Leeds LS2 9JT, U.K.; 4 School of Food Science and Nutrition, University of Leeds, Woodhouse Lane, Leeds LS2 9JT, U.K.; 5 Science for Life Laboratory, Department of Women's and Children's Health, Karolinska Institutet, 17165 Solna, Sweden

**Keywords:** biophysics, confocal microscopy, lattice light sheet microscopy, single molecule localization microscopy, super-resolution imaging, widefield microscopy

## Abstract

Advanced imaging is key for visualizing the spatiotemporal regulation of immune signaling which is a complex process involving multiple players tightly regulated in space and time. Imaging techniques vary in their spatial resolution, spanning from nanometers to micrometers, and in their temporal resolution, ranging from microseconds to hours. In this review, we summarize state-of-the-art imaging methodologies and provide recent examples on how they helped to unravel the mysteries of immune signaling. Finally, we discuss the limitations of current technologies and share our insights on how to overcome these limitations to visualize immune signaling with unprecedented fidelity.

## Introduction

**Figure 1.**
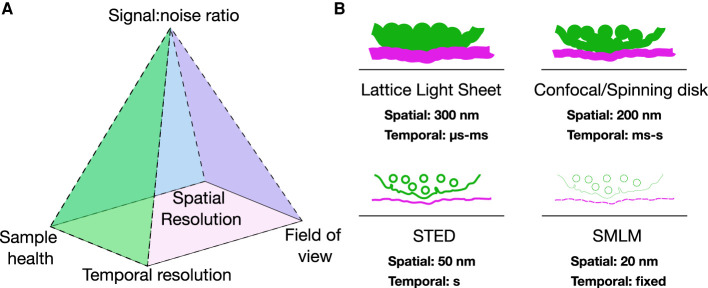
Demands for different microscopy techniques. (**A**) Pyramid of frustration in microscopy. One single imaging technique cannot cover all axes of organismal, cellular and molecular imaging. Compromises from certain aspects are common to gain in others. (**B**) Imaging modalities discussed in this review and their comparison in spatiotemporal resolution.

**Figure 2.**
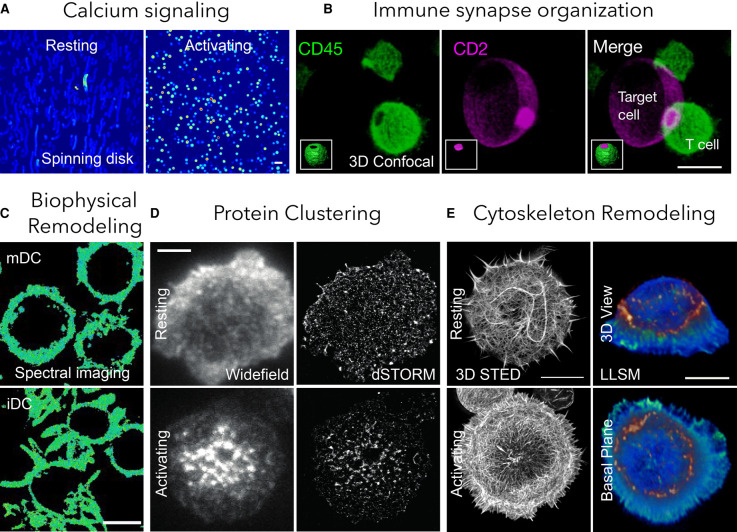
Potential applications of different imaging modalities in immunity. (**A**) Calcium signaling with spinning disk microscopy [116] where bright spots show calcium flux (scale bar 20 µm); (**B**) 3D immune synapse with confocal microscopy [146] where magenta is CD2 on target synthetic cells and green is CD45 in T cells (scale bar 10 µm); (**C**) biophysical imaging with spectral imaging combined with smart probes [103] where immature dendritic cells (iDCs) show lower membrane fluidity than mature dendritic cells (mDCs) (scale bar 10 µm); (**D**) protein clustering with SMLM [48] (scale bar 3 µm); (**E**) cytoskeleton imaging with STED and LLSM [33] (scale bar 10 µm).

Immune signaling processes occur over broad spatial and temporal scales. The spatial reorganization of immune signaling ranges from large micron-sized patterns, termed supramolecular activation clusters (SMACs) to signaling micro-clusters and nanoclusters below the resolution limit of conventional light microscopy [1,2]. The temporal events can span from microsecond exploratory interactions to stable cell–cell contacts lasting over minutes. One single imaging technique cannot cover all spatial and temporal scales simultaneously and it is necessary to compromise in certain aspects to gain in others (Figure 1). Therefore, different modes of cellular imaging are used to enable visualization of specific molecules and to elucidate their interplay, structure and the resulting biological signaling pathways without significant perturbation of the fragile machinery of the immune response. In this review, we summarize recent technologies that provide insight into the molecular details of immune cell signaling. We cover dynamic imaging for long-term visualization, super-resolution microscopy beyond the diffraction limit and biophysical imaging for measuring collective cellular properties. By showcasing recent examples of how these techniques have been used to unravel the molecular details of immune signaling, we aim to provide a general understanding of this unique toolbox for immunologist (Figure 2).

## Dynamic imaging of immune signaling

The triggering of the T-cell receptor (TCR) occurs within a few seconds of cell–cell contact [1], which involves reorganization of membrane proteins over 20–100 nm [3] and the fast motion of finger-like microvilli structures [4,5] that continuously change in shape [6]. These conditions all together present highly technical imaging challenges. As every microscopy technique has their own advantages and limitations (Figure 1), the dynamic imaging of immune signaling processes has been approached with a variety of microscopy techniques.

### Imaging the interface

The interface between a glass coverslip and immune cells presents an attractive platform for imaging signaling processes occurring at the plasma membrane. Total internal reflection fluorescence microscopy (TIRFM) can be applied to create an evanescent wave that penetrates 100–200 nm into the cell [7], enabling high-contrast imaging of the glass-cell interface. This has been used to visualize formation of SMACs [8], nanoscale reorganization of T-cell membrane proteins [3] and formation of microclusters in B cells [9] and T cells [8]. Naturally, the high contrast of TIRFM lends itself to single-molecule imaging of membrane proteins [10] (covered in the subsequent super-resolution section). While TIRFM achieves high axial resolution, it is limited to 2D with diffraction-limited resolution. By controlling the incident angle of the laser beam reflected off the coverslip glass, the penetration depth of the evanescent beam can be varied to gain 3D information. Such variable-angle (VA)-TIRFM [11] has been used to study the distribution and the size of membrane proteins and membrane topography [3,11]. Like with TIRFM, the topography of the glass-membrane interface can also be measured by taking advantage of the refractive index change between the cell membrane and the cell media. Interference reflection microscopy (IRM) is a label-free technique that has been applied to study adhesion of immune cells to model antigen-presenting cell surfaces [3,13]. By coating the surface with a reflective metal and varying the incident angle to enable scanning angle interference microscopy (SAIM), it is also possible to perform axial measurements with nm precision [14]. This precision is key for discerning topographical variations in membrane protein distributions and it has been applied to reveal that TCR microclusters are enriched closer to the activating interface as opposed to bulk TCR [6].

These methods all attempt to model a cell–cell interface, typically by coating the coverslip with a supported lipid bilayer (SLB) that incorporates membrane proteins. However, this presents challenges as the physiological relevance of SLBs can be questioned due to unknown role of the glycocalyx [15], unexpected cell activation [13,16] and the effect of the hard glass on mechanosensitive cells [17]. To minimize artifacts, immune signaling should ideally be studied in 3D and preferably at the intercellular interface.

### Dynamic 3D imaging

Fluorescence images of an immunological synapse (IS) were first acquired using confocal microscopy in the late 90s [18,19]. Commercial spinning disk confocal microscopes routinely achieve diffraction-limited resolution (lateral: 200 nm, axial: 500 nm) with video rate 2D imaging (20 Hz) or slightly slower 3D imaging (∼1 Hz). Most immune signaling studies have relied on this imaging technique with examples including demonstration of finger-like structures breaking through the cell membrane glycocalyx [20], the release of cell-killing attack particles [21] or calcium influx [13]. The main disadvantages of confocal microscopy are the limited speed and the propensity for photobleaching (Table 1). Multi-point detector confocal techniques such as Airyscan super-resolution imaging [22] have been able to effectively double the spatial resolution, which has enabled dynamic 2D imaging to resolve B cell synapse formation [23]. However, the ∼10-fold reduction in speed has been prohibitive for dynamic 3D imaging of larger structures, such as the cell–cell interface.

**Table 1:** Comparison of the available techniques to investigate immune signaling processes (fps: frame per second)

Technique	XY res (nm)	Z res (nm)	Temporal (fps)	Limitation	Advantages	Typical use
Total internal reflection fluorescence (TIRF) microscopy	250	100	100	Only the interface with coverslip	High contrast	Synapse formation, single-molecule dynamics
Variable angle (VA)-TIRF microscopy	250	10	100	Only the interface with coverslip	Z resolution++	Cell topography
Confocal	250	500	20	Photobleaching	Simplicity	Cell structure, interactions
Airyscan	125	250	5	Photobleaching	Resolution	Cell structure, interactions
Stimulated emission depletion (STED) microscopy, ground state depletion (GSD) microscopy	20	50	1	Toxicity, Adequate dyes	Instant super-resolution	Live cell super-resolution, Combination with single molecule
Instant structural illumination microscopy (SIM)	125	250	100	Photobleaching	Speed, resolution	Dynamic cell–cell interactions
Lattice Light-sheet microscopy	250	500	100	Setup complexity	Gentle and fast imaging	Dynamic cell–cell interactions
Lattice SIM	125	250	20	Setup complexity	Gentle and high resolution imaging	Dynamic cell–cell interactions
Single molecular localization microscopy (SMLM)	15	15	-	Chemically fixed cells, Elaborate data analysis	Axial and lateral resolution	Local and global distribution of molecules
Minimal photon fluxes (MINFLUX) microscopy	2 (20)	2 (20)	- (100 µs)	Chemically fixed cells, Elaborate data analysis	Axial and lateral resolution	Cell structure, interactions, Localization of molecules (tracking)

Widefield fluorescence techniques utilize cameras to overcome scanning-induced speed limitations of confocal microscopy, but with reduced axial sectioning abilities. Structured illumination microscopy (SIM) is a widefield super-resolution microscopy technique that uses interference patterns to acquire multiple patterned fluorescence images from varying directions. These sequences then enable reconstruction of super-resolution images. This practically doubles resolution in all dimensions to achieve 125 nm and 350 nm resolutions in lateral and axial dimensions, respectively. In terms of immune signaling, most dynamic imaging studies to date have been performed in 2D [23]. Combination of SIM with advanced analysis techniques such as image correlation spectroscopy has enabled analysis of fast membrane protein dynamics [24]. With instant SIM [25], sub-second cellular 3D imaging has now become possible, but photobleaching problems are still prevalent, preventing long-term imaging.

Confocal, SIM and epifluorescence microscopy are all prone to photobleaching as most of the sample is continuously exposed to laser excitation. To achieve gentler imaging, light-sheet microscopy (also known as selective-plane illumination microscopy) techniques have been developed, which selectively excite fluorophores within the focal plane of the sample. This combines the advantages of TIRFM and off-surface methods to enable 3D imaging with axial sectioning and high contrast due to reduced out-of-focus excitation. However, the difficulty in applying light-sheet microscopy to bioimaging has always involved the complexity of introducing the light sheet into the sample. Given the need for high numerical aperture objective lenses to visualize signaling processes, a variety of complex solutions have been developed including tilted objective lenses [16,26], special sample chambers [27], prisms [28], and reflective surfaces [29–31].

A major improvement came with the development of lattice light-sheet microscopy (LLSM) that offered optical sectioning (1 µm full width at half maximum sheet thickness) over a large field-of-view (50 µm) [6,32]. This imaging modality achieves high resolution (230 nm xy and 370 nm z, or 150 nm xy and 280 nm z in SIM mode) and high-speed imaging while minimizing phototoxicity and photobleaching effects. Over the past seven years, this technique has developed into one of the most promising techniques for imaging dynamic interactions between immune cells in 3D, for example revealing the topological changes taking place during T cell IS formation [32], how finger-like cellular structures search for antigens [6] and how the actin cytoskeleton facilitates immune activation [33,34] (Figure 2). Its combination with adaptive optics has now enabled dynamic 3D imaging of immune cells in living tissue [35]. Recently, high numerical aperture oblique plane microscopy [36] has been implemented to enable high-resolution (300 nm xy and 600 nm z) single-objective light-sheet microscopy for imaging immune cell interactions [37]. This is currently the most accessible choice for immunology labs building their own microscopes. LLSM for common sample geometries has also become accessible through commercial solutions that can achieve multi-color imaging with 700 nm optical sectioning thickness over a field of view of 30 × 300 µm at a speed of 400 frames per second.

Dynamic diffraction-limited 3D imaging of immune cells has mostly been solved with above introduced techniques, although there are needs in terms of imaging deeper into tissue and simplifying setups for increased accessability. While a great deal can be learned, understanding of immune signaling processes on the molecular scale requires nanoscale resolution, thus the field is in need to combine its efforts with super-resolution imaging techniques while maintaining the high temporal resolution offered by the dynamic 3D imaging techniques.

## Super-resolution imaging of immune signaling

Super-resolution imaging modalities paved the road to investigate immune signaling down to individual signal events initiated or mediated by single receptors, kinases, phosphatases, adaptor molecules or other contributing biomolecules. Nowadays commercial availability of super-resolution microscopes facilitates their active utilization to study cellular processes, such as immune signaling, on the nanometer scale. Common to all super-resolution modalities discussed in this section is the suppression of fluorescence emission from labels coupled to the biomolecule of interest to achieve spatial resolutions far below the classical resolution limit of light microscopy (∼250 nm).

In stochastic and camera-based approaches like (direct) stochastic optical reconstruction microscopy ((d) STORM) [38,39], (fluorescence) photoactivated localization microscopy ((f)PALM) [40,41], points accumulation for imaging in nanoscale topography (PAINT) [42], or ground state depletion microscopy followed by individual molecule return (GSDIM) [43], fluorophores are switched between visible and invisible states (for how this is achieved see ref [44]). Conditions are chosen such that most molecules are invisible and hence omitted from imaging. As a result, average distances between visible molecules become larger than the resolution limit of the microscope. Individual molecules can now be computationally localized with precision only limited by the number of detected photons and background noise [45,46], and typically reach values of a few nanometers. By recording tens of thousands of such images and subsequent localization of all single molecule signals, a super-resolution image of molecular positions can be reconstructed. These molecular maps are often consulted to determine the local and global distribution of molecules on the plasma membrane. Examples range from the TCR [47–49], the adaptor LAT [47,50], the kinases Lck [51,52] and ZAP70 [53], and many others, to the complex interplay of individual components within the TCR [54], BCR [55,56] or NK [57] signaling pathways. Numerous mathematical approaches are currently available for the analysis of localization maps (an overview can be found in ref [44]). For most examples provided, the interface between a functionalized glass and an immune cell was studied by using TIRFM as imaging modality. Beside the gain in contrast, the vicinity of fluorescently labeled molecules to glass allows for using the supercritical angle fluorescence as a parameter for determining the distance of fluorophores to the glass surface with nanometer precision also in the axial direction [58,59]. This combination enabled to study the topography of the immunological synapse at isotropic localization precision below 15 nm [49] (Figure 2).

Recording thousands of frames for super-resolution imaging takes minutes to hours, rendering the observation of biomolecular dynamics challenging [60]. Therefore, most studies are still performed on chemically fixed cells. One possibility to overcome residual motion [61] and potential artifacts described for chemical fixation is to conduct super-resolution microscopy experiments at cryogenic temperatures. Although this approach was not applied to study immune signaling yet, current technical developments and first results look promising for using cryo-super-resolution for the routine detection and characterization of biomolecular assemblies on the sub-nanometer length scale [62].

In contrast with these ‘single molecule localization microscopy' (SMLM) techniques, stimulated emission depletion (STED) [63] or ground state depletion (GSD) [64], use a deterministic approach to suppress unwanted fluorescence at specific positions. By combining an excitation laser with a second laser, which switches fluorescence off everywhere but in the center of the activation laser, nanoscopic activation spots are created with sizes much smaller than the diffraction limit of ∼250 nm. Scanning of both lasers over the sample and collecting the fluorescence emission by a point detector allows the reconstruction of a super-resolution image. Although the resolution achieved is in most cases worse compared with SMLM, STED is faster and can be used for live cell imaging, for example, to image directly cytoskeletal actin dynamics during IS formation [33] (Figure 2).

Of note, long before the advent of super-resolution imaging, the high precision in localizing individual signals was utilized in single-particle or single-molecule tracking (SPT, SMT) to extract information about locations, kinetics, dynamics and interactions of individual molecule. The basic concept is to directly observe the motion of individual signals in live cell experiments and to extract the aforementioned information from trajectories generated from localized particles or molecules [65]. High-speed SPT and SMT allowed the detection of plasma membrane compartmentalization via hop-diffusion of MHC class II molecules [66] far below the typical resolution limit of light microscopy. The direct observation of interaction dynamics between TCR and peptide-MHC [67,68], or between TCR and ZAP 70 [69] on the millisecond to seconds timescale was facilitated by tracking experiments. Combining SPT/SMT with single-molecule Förster resonance energy transfer (FRET), multicolor and 3D imaging, is hence a powerful approach for live cell studies, however, with one major caveat: the density of fluorescently labeled molecules needs to be low enough for unambiguous tracking. Only if combined with photo-bleaching, SMT can be applied to characterize the entire population of molecules, as shown for characterizing the oligomeric state of the TCR [70].

Super-resolution techniques immensely contribute to our understanding of immune signaling by identifying spatiotemporal positions of individual key molecules on the nanometer-scale. However, the precise mode of action of these molecules within the signaling process needs to be addressed separately. One parameter gaining increasing attention in the field of immunology is the exertion of or response to mechanical forces [71].

## Imaging forces in immune signaling

The structural plasticity of immune cells facilitated by cytoskeletal remodeling has been shown to exert a substantial amount of force on neighboring cells, on the extracellular matrix, and to the components of the IS. It changes the conformation of individual molecules, initiates cluster formation, and alters bond kinetics and biophysical properties such as membrane stiffness. Remodeling of the cytoskeleton, similar to cell spreading and motility, results in cycles of pushing and pulling [72,73], which coordinate and terminate different signaling cascades. To understand the role of mechanical forces, novel techniques have to be used and developed.

While we reviewed techniques to probe mechanical forces elsewhere [74], the field has received significant attention with a plethora of new discoveries. There are several methods available to probe the involvement of tensile load application during immune cell activation. For T cells, with astonishing discriminative power, TCR–pMHC interactions are tested by mechanical load application, leading to specific activation [75]. The kinetic proofreading model, being based on TCR-proximal signaling triggered through TCR–pMHC bond lifetimes, was first used to explain the discriminative power [76], with raising attention on mechanical load application [77]. Single-molecule FRET imaging revealed that lifetimes of TCR–pMHC bonds were significantly altered compared to in solution [67], with cellular dynamics mediated by the actin cytoskeleton as a potential reason. The TCR–pMHC unbinding under force was found to have characteristics of both catch bond [72,78] and slip bond [79] behavior, using standard surface plasmon resonance studies as well as biomembrane force probes, where force is measured through reading out the deformation of cells as a force sensor [80]. Recent discoveries have identified a mechanical force range in the piconewton (pN) regime. Note that a force of 10 pN is equal to the weight of ∼10 T-cells or 1 million corona viruses on a surface and it is sufficient to rupture bonds and alter conformations of proteins. The impact of such small forces on immune cell signaling is described in the following.

Besides the structural dynamics of IS formation [81], to trigger T-cell signaling, single TCRs were strikingly shown to be transported to the center of the IS via the cytoskeleton with resulting shear forces of ∼10 pN measured by optical tweezers [82]. Furthermore, micropipette experiments with TCR-ligand coated beads revealed T-cell signaling in the form of calcium release. Beads, therefore, were retracted after T cell engagement [83]; when beads were moved tangentially with respect to the T-cell membrane activation occurred more likely [82], proving that the direction of applied forces matters [76,77]. In a different set of experiments using deformable substrates it was shown that T cells can sense the elastic properties of their surroundings [84]. This technique, named traction force microscopy (TFM) (for review of the techniques in the context of immune signaling please see ref [74]) enabled the study of pulling forces using micropillar assays [85] and hydrogel bound microbeads [86]. Also for B cells, TFM was used to record force application of cells to their surrounding during the activation process [87], suggesting that there is active sensing between groups of immune cells. Most recently, TFM has been combined with 2D TIRF-SIM [88] to correlate force readout with cytoskeletal remodeling and identified the importance of even individual microvilli for the force-mediated T-cell activation process [89].

To detect forces on the single TCR–pMHC bond level, DNA-based tension sensors were instated, suggesting forces as high as 12 pN for ligand-bound TCRs [90] on glass. In contrast, forces as high as 4.7 pN were measured on gold particles attached to a supported lipid bilayer [91]. Tuning the maximal force via the DNA tension sensor enabled ZAP70 recruitment to the engaged TCRs as a secondary signaling molecule [90]. However, DNA tension sensors are hard to predict for ensemble measurements as it is unclear if they are arranged in parallel or zipper-like configuration. Furthermore, it is difficult to estimate how many molecules are engaged simultaneously. Moreover, the unzipping force is strongly loading-rate [92] and duration of applied force [93] dependent. As an alternative, FRET tension sensors have been established for focal adhesions [94] with the perspective of time dependence studies of TCR — pMHC interaction and involved forces [71].

## Imaging biophysics of immune signaling

Cellular biophysical properties such as cellular deformability, stiffness, membrane fluidity, viscosity and tension differ for different cells, cellular states and diseases [95,96]. Such properties play crucial roles in immune cell activation, differentiation, and function, therefore methodologies to accurately measure these properties are needed. Biophysical properties are usually measured with advanced imaging technologies in combination with smart probes. Membrane fluidity can be measured using environment-sensitive probes whose emission spectra change depending on the physicochemical properties of the environment. Polarity-sensitive probes, for instance, exhibit more blue-shifted emission in rigid membranes compared with fluid membranes. To quantitatively evaluate the membrane fluidity, this spectral shift can be detected by widefield, confocal or super-resolution imaging [97]. Membrane viscosity and tension can be measured with molecular rotors [98] or push-and-pull probes [99] which change their fluorescence lifetime depending on the viscosity or the tension in the environment. These probes require sensitive detectors with single-photon counting capacity (so called ‘time-correlated single-photon counting' detectors) attached to imaging units. Measuring cellular stiffness, on the other hand, does not require fluorescent probes. Atomic force microscopy has been used extensively to measure the stiffness of cells [100], however, it is limited by its low throughput (i.e. one cell at a time). Recent real-time deformability technologies rely on microfluidics devices measuring the cell shapes using brightfield light [101]. With this technology, adaptability of the shape as a function of physical resistance is used to obtain the stiffness of thousands of cells in a short time [102].

Immune cells undergo extensive biophysical remodeling during their lifetime which is necessary for their function. A recent example by Lühr et al. [103] using environment-sensitive probes, showed that monocyte-derived dendritic cells change their membrane fluidity and cellular stiffness during cellular maturation (Figure 2). In a similar example, Matias et al. [104] demonstrated that regulatory T-cell differentiation is correlated with biophysical remodeling. Importantly, such biophysical properties can influence the migration potential [105] and signaling potency of immune cells [106], which are crucial factors for immune responses, such as during inflammation and infection [107]. Apart from remodeling their own biophysical properties, immune cells can also sense the biophysical cues from their target cells. A recent example by Tello-Lafoz et al. [108] suggested that biophysical vulnerabilities in cancer cells can be recognized and utilized by immune cells. The stiffness of target cells, for example, is a crucial factor for immune cell recognition and activation [17,109,110].

Biophysical properties of the IS attracted particular attention in the last decade. There is significant evidence suggesting a major role of biophysical properties of IS as regulating factors for signaling [111,112]. Owen et al. [113] showed that the IS has higher membrane order compared with the rest of the plasma membrane. Recent studies suggested that this higher membrane order in the IS serve as a protection mechanism against self-killing [114,115]. Moreover, electrical charge in the IS might also be different from the rest of the PM due to the exocytosis of secretory granules with negatively charged lipids. This difference in charge can help fulfil IS function and, similar to membrane order, protect the IS against its own toxins [115]. Jung et al. [100] showed that stiffness of T cell changes upon IS formation which adds another regulation mechanism for IS activity [109]. Size of the molecules at the IS constitutes an additional parameter in the IS biophysical landscape. Tall molecules are excluded from the tight contact between the immune and target cells, which can be a biophysical regulation mechanism for activation of various immune cells [3,116,117]. Finally, diffusion and aggregation of the molecules at the IS have been suggested as a crucial biophysical mechanism to fine-tune signaling [4,118]. The IS has been an important target for therapies such as immunotherapy and the accumulating evidence on the biophysical aspects of the IS shows that such collective biophysical properties should be considered for therapies targeting the IS [119].

## Limitations and common artifacts while imaging immune signaling

While advanced imaging techniques reveal new important and complex details about the immune response, they come with technical, biological, and methodological limitations. In many applications mentioned before, the complexity of the biological systems was kept as low as possible to allow for high-contrast imaging. For example, imaging was performed on cells interacting with 2D surfaces to enable TIRFM, or simple cell–cell interactions in solution were observed using (lattice) light-sheet microscopy. Looking directly at immunological processes occurring in tissue, e.g. lymph nodes, demands new imaging strategies or modifications of existing techniques. Recently, the multiscale reorganization of TCRs during the *in vivo* immune response was imaged in lymph nodes by light sheet dSTORM and SIM [120], demonstrating the suitability of advanced techniques also for more complex systems by combining imaging concepts. Other limitations to overcome are the spatial resolution in dynamic imaging — or analogously — the temporal resolution in super-resolution imaging (Figure 1). While current and future developments will address these challenges, the achieved signal-to-noise ratio depends ultimately on the photophysics of used organic dyes, fluorescent proteins or other fluorescent markers. For all techniques, the number of detected photons will set the limit for achievable temporal and spatial resolution. In addition, the complex and not fully understood photophysics of fluorophores might lead to misinterpretation, as was recently demonstrated for the nanoscale organization of TCR [48] or Lck [52]. Software taking, for example, the experimentally determined blinking properties of fluorophores into account [121] or elaborate experimental strategies (e.g. by titrating the label [52], temporal accumulation analysis [122] or using spectrally distinct probes [123]) might overcome these artifacts.

Limits are also set for the resolution of mechanical forces, where probes and methods sensing a high dynamic force range are still missing. Also, the ultimate aim of a force read-out for individual molecules within the whole IS will demand the combination of various imaging techniques, as was shown in first attempts by increasing the spatiotemporal resolution [88] or addressing forces in 3D [124].

Another major limitation to overcome is the handling and storage of large amounts of data generated during image acquisition. New concepts to increase the computational power for data analysis will have to be instated. The increasing complexity of microscopy systems and generated data will have to be matched by well trained and skilled operators and machine learning/artificial intelligence-based analysis algorithms, respectively.

## Future

Immune signaling has been imaged at high speed [32], high resolution [49], multispectrally [125] and deep inside living tissue [35]. The main challenges in the field relate to breaking the pyramid of frustration (Figure 1) to combine these achievements and push towards real-time super-resolution imaging in 3D, ideally while imaging multiple targets and avoiding phototoxicity/photobleaching. Such capability would enable unprecedented insight into how complex players of the immune system coordinate to sustain basic signaling functions. Furthermore, the increased speed and resolution offer opportunities of being combined with force- and signaling-fluorescence assays to understand how these mechanisms correlate with the organization of cellular components.

Dynamic techniques (<1 s per image) are constantly pushing the spatial resolution limits. Parallelized RESOLFT, which relies on photoswitchable fluorescent proteins, now achieves 80 nm 3D resolution in entire cells at 1–2 frames per second [126]. Light-sheet has previously been combined with RESOLFT to achieve <100 nm sectioning [127] and the recent combination of light sheet with 4Pi microscopy achieves <100 nm sectioning with conventional fluorophores [128].

High-resolution SMLM techniques (<10 nm) have traditionally suffered from particularly slow imaging speed. However, improvements in analysis pipelines through deep-learning approaches [129,130], optimization of imaging parameters [131] and novel labeling strategies [132] make dynamic 3D super-resolution imaging a future inevitability. Notably, the recently introduced MINFLUX [133] offers unprecedented spatial and temporal resolution, which will aid in this endeavor. In addition, minimal emission fluxes reduce the probability of fluorophore photobleaching and hence allow tracking of individual molecules over long time periods. Its potential parallelization, as previously done for STED [134] and RESOLFT [126], holds great potential for future dynamic imaging applications.

Another aspect holding the field back is availability of state-of-the-art techniques and analysis tools. As mentioned earlier, it took ∼7 years to develop a turn-key commercial LLSM. Fortunately, the microscopy field has been embracing democratization of designs and low-cost solution to bring new techniques to the masses. 3D printed microscopes [135,136] offer great affordability and flexibility. Deep-learning approaches for analysis and denoising are readily available for implementation on online platforms [137]. Complete low-cost designs are available for confocal [138], widefield [139,140] and SMLM [141].

While dynamic imaging is key, there is much left to be learned from static approaches. Expansion microscopy has recently been used to map out receptors on T cells [12], which has the potential to be applied to most immune cells. The structure of the IS is particularly paramount, where SMLM and expansion microscopy offer great potential in visualizing receptor organization. Nevertheless, it is important to acknowledge and resolve the challenges involved both with fixation [142] and expansion microscopy [143] and an exciting solution may lie in the application of cryo-SMLM that can overcome these limitations [144].

Despite these technical developments, there is something inherently difficult about imaging cell–cell interactions and synapse formation and there are only a handful of studies out there taking advantage of the latest methodologies. This partially stems from the move into dynamic 3D super-resolution imaging being far from trivial. The relatively fast restructuring of the cell membrane and the reorganization of membrane proteins present a complex challenge involving multiple unanswered questions, even with the availability of high-speed high-resolution techniques. Where exactly is the membrane or glycocalyx? Where are the proteins in relation to the membrane? Is the cell currently signaling? Is the cell under stress? Furthermore, the basic definition of detecting signaling is itself challenging: does it correspond to calcium flux, expression of certain proteins or formation of signaling complexes? Complementary labeling approaches have the potential to define where the interactions occur, which can provide a helpful cue [145], but there is a large scope of applying such labeling or biochemical approaches. Nevertheless, one thing is clear: to fundamentally change our understanding of immune signaling through these new visualizations, it will be necessary to develop novel analytical approaches that can quantify multispectral 3D data and correlate it with the highly dynamic complex topography of the cell membrane.

## Perspectives

- Advanced imaging is key for visualizing the spatiotemporal regulation of immune signaling.
- We summarize state-of-the-art imaging methodologies and provide recent examples on how they helped to unravel the mysteries of immune signaling.
- We discuss the limitations of current technologies and share insights on how to overcome these limitations to visualize immune signaling with unprecedented details.

## Open Access

Open access for this article was enabled by the participation of Karolinska Institute in an all-inclusive *Read & Publish* agreement with Portland Press and the Biochemical Society.

## Abbreviations

FRET: Förster resonance energy transfer
GSD: ground state depletion
LLSM: lattice light-sheet microscopy
SIM: structured illumination microscopy
SMACs: supramolecular activation clusters
SMLM: single molecule localization microscopy
STED: stimulated emission depletion
TCR: T cell receptor
TFM: traction force microscopy
TIRFM: total internal reflection fluorescence microscopy
VA: variable-angle

## References

[1] Huse, M., Klein, L.O., Girvin, A.T., Faraj, J.M., Li, Q.-J., Kuhns, M.S. et al. (2007) Spatial and temporal dynamics of T cell receptor signaling with a photoactivatable agonist. Immunity 27, 76–88 10.1016/j.immuni.2007.05.017 17629516

[2] Kuhns, M.S. and Davis, M.M. (2012) TCR signaling emerges from the Sum of many parts. Front. Immunol. 3, 159 10.3389/fimmu.2012.00159 22737151 PMC3381686

[3] Chang, V.T., Fernandes, R.A., Ganzinger, K.A., Lee, S.F., Siebold, C., McColl, J. et al. (2016) Initiation of T cell signaling by CD45 segregation at “close contacts.”. Nat. Immunol. 17, 574–582 10.1038/ni.3392 26998761 PMC4839504

[4] Chen, K.Y., Jenkins, E., Körbel, M., Ponjavic, A., Lippert, A.H., Santos, A.M. et al. (2021) Trapping or slowing the diffusion of T cell receptors at close contacts initiates T cell signaling. Proc. Natl Acad. Sci. U.S.A. 118, e2024250118 10.1073/pnas.2024250118 34526387 PMC8488633

[5] Aramesh, M., Stoycheva, D., Sandu, I., Ihle, S.J., Zünd, T., Shiu, J.-Y. et al. (2021) Nanoconfinement of microvilli alters gene expression and boosts T cell activation. Proc. Natl Acad. Sci. U.S.A. 118, e2107535118 10.1073/pnas.2107535118 34599101 PMC8501847

[6] Cai, E., Marchuk, K., Beemiller, P., Beppler, C., Rubashkin, M.G., Weaver, V.M. et al. (2017) Visualizing dynamic microvillar search and stabilization during ligand detection by T cells. Science 356, eaal3118 10.1126/science.aal3118 28495700 PMC6364556

[7] Axelrod, D. (1989) Total internal reflection fluorescence microscopy. Methods Cell Biol. 30, 245–270 10.1016/S0091-679X(08)60982-6 2648112

[8] Varma, R., Campi, G., Yokosuka, T., Saito, T. and Dustin, M.L. (2006) T cell receptor-proximal signals are sustained in peripheral microclusters and terminated in the central supramolecular activation cluster. Immunity 25, 117–127 10.1016/j.immuni.2006.04.010 16860761 PMC1626533

[9] Davis, R.E., Ngo, V.N., Lenz, G., Tolar, P., Young, R.M., Romesser, P.B. et al. (2010) Chronic active B-cell-receptor signalling in diffuse large B-cell lymphoma. Nature 463, 88–92 10.1038/nature08638 20054396 PMC2845535

[10] Douglass, A.D. and Vale, R.D. (2005) Single-molecule microscopy reveals plasma membrane microdomains created by protein-protein networks that exclude or trap signaling molecules in T cells. Cell 121, 937–950 10.1016/j.cell.2005.04.009 15960980 PMC2851620

[11] Cardoso Dos Santos, M., Déturche, R., Vézy, C. and Jaffiol, R. (2016) Topography of cells revealed by variable-Angle total internal reflection fluorescence microscopy. Biophys. J. 111, 1316–1327 10.1016/j.bpj.2016.06.043 27653490 PMC5034310

[12] Jung, Y., Riven, I., Feigelson, S.W., Kartvelishvily, E., Tohya, K., Miyasaka, M. et al. (2016) Three-dimensional localization of T-cell receptors in relation to microvilli using a combination of superresolution microscopies. Proc. Natl Acad. Sci. U.S.A. 113, E5916–e5924 10.1073/pnas.1604894113 27647916 PMC5056101

[13] Santos, A.M., Ponjavic, A., Fritzsche, M., Fernandes, R.A., de la Serna, J.B., Wilcock, M.J. et al. (2018) Capturing resting T cells: the perils of PLL. Nat. Immunol. 19, 203–205 10.1038/s41590-018-0048-8 29476188 PMC7612954

[14] Paszek, M.J., DuFort, C.C., Rubashkin, M.G., Davidson, M.W., Thorn, K.S., Liphardt, J.T. et al. (2012) Scanning angle interference microscopy reveals cell dynamics at the nanoscale. Nat. Methods 9, 825–827 10.1038/nmeth.2077 22751201 PMC3454456

[15] Fernandes, R.A., Ganzinger, K.A., Tzou, J.C., Jönsson, P., Lee, S.F., Palayret, M. et al. (2019) A cell topography-based mechanism for ligand discrimination by the T cell receptor. Proc. Natl Acad. Sci. U.S.A. 116, 14002–14010 10.1073/pnas.1817255116 31221762 PMC6628812

[16] Ponjavic, A., McColl, J., Carr, A.R., Santos, A.M., Kulenkampff, K., Lippert, A. et al. (2018) Single-Molecule light-Sheet imaging of suspended T cells. Biophys. J. 114, 2200–2211 10.1016/j.bpj.2018.02.044 29742413 PMC5961759

[17] Saitakis, M., Dogniaux, S., Goudot, C., Bufi, N., Asnacios, S., Maurin, M. et al. (2017) Different TCR-induced T lymphocyte responses are potentiated by stiffness with variable sensitivity. eLife 6, e23190 10.7554/eLife.23190 28594327 PMC5464771

[18] Monks, C.R., Freiberg, B.A., Kupfer, H., Sciaky, N. and Kupfer, A. (1998) Three-dimensional segregation of supramolecular activation clusters in T cells. Nature 395, 82–86 10.1038/25764 9738502

[19] Davis, D.M., Chiu, I., Fassett, M., Cohen, G.B., Mandelboim, O. and Strominger, J.L. (1999) The human natural killer cell immune synapse. Proc. Natl Acad. Sci. U.S.A. 96, 15062–15067 10.1073/pnas.96.26.15062 10611338 PMC24773

[20] Sage, P.T., Varghese, L.M., Martinelli, R., Sciuto, T.E., Kamei, M., Dvorak, A.M. et al. (2012) Antigen recognition is facilitated by invadosome-like protrusions formed by memory/effector T cells. J. Immunol. 188, 3686–3699 10.4049/jimmunol.1102594 22442443 PMC3324627

[21] Bálint, Š., Müller, S., Fischer, R., Kessler, B.M., Harkiolaki, M., Valitutti, S. et al. (2020) Supramolecular attack particles are autonomous killing entities released from cytotoxic T cells. Science 368, 897–901 10.1126/science.aay9207 32381591 PMC7116847

[22] Huff, J. (2015) The airyscan detector from ZEISS: confocal imaging with improved signal-to-noise ratio and super-resolution. Nat. Methods 12, i–ii 10.1038/nmeth.f.388

[23] Bolger-Munro, M., Choi, K., Scurll, J.M., Abraham, L., Chappell, R.S., Sheen, D. et al. (2019) Arp2/3 complex-driven spatial patterning of the BCR enhances immune synapse formation, BCR signaling and B cell activation. eLife 8, e44574 10.7554/eLife.44574 31157616 PMC6591008

[24] Ashdown, G.W., Cope, A., Wiseman, P.W. and Owen, D.M. (2014) Molecular flow quantified beyond the diffraction limit by spatiotemporal image correlation of structured illumination microscopy data. Biophys. J. 107, L21–L23 10.1016/j.bpj.2014.09.018 25418107 PMC4223199

[25] York, A.G., Chandris, P., Nogare, D.D., Head, J., Wawrzusin, P., Fischer, R.S. et al. (2013) Instant super-resolution imaging in live cells and embryos via analog image processing. Nat. Methods 10, 1122–1126 10.1038/nmeth.2687 24097271 PMC3898876

[26] Gustavsson, A.-K., Petrov, P.N., Lee, M.Y., Shechtman, Y. and Moerner, W.E. (2018) Tilted light sheet microscopy with 3D point spread functions for single-Molecule super-Resolution imaging in mammalian cells. Proc. SPIE Int. Soc. Opt. Eng. 10500, 105000M 10.1117/12.2288443 PMC5906058 29681676

[27] Ritter, J.G., Veith, R., Siebrasse, J.-P. and Kubitscheck, U. (2008) High-contrast single-particle tracking by selective focal plane illumination microscopy. Opt. Express 16, 7142–7152 10.1364/OE.16.007142 18545417

[28] Hu, Y.S., Zhu, Q., Elkins, K., Tse, K., Li, Y., Fitzpatrick, J.A.J. et al. (2013) Light-sheet Bayesian microscopy enables deep-cell super-resolution imaging of heterochromatin in live human embryonic stem cells. Opt. Nanoscopy 2, 7 10.1186/2192-2853-2-7 27795878 PMC5082751

[29] Gebhardt, J.C.M., Suter, D.M., Roy, R., Zhao, Z.W., Chapman, A.R., Basu, S. et al. (2013) Single-molecule imaging of transcription factor binding to DNA in live mammalian cells. Nat. Methods 10, 421–426 10.1038/nmeth.2411 23524394 PMC3664538

[30] Galland, R., Grenci, G., Aravind, A., Viasnoff, V., Studer, V. and Sibarita, J.-B. (2015) 3D high- and super-resolution imaging using single-objective SPIM. Nat. Methods 12, 641–644 10.1038/nmeth.3402 25961414

[31] Ponjavic, A., Ye, Y., Laue, E., Lee, S.F. and Klenerman, D. (2018) Sensitive light-sheet microscopy in multiwell plates using an AFM cantilever. Biomed. Opt. Express 9, 5863–5880 10.1364/BOE.9.005863 31065399 PMC6490997

[32] Chen, B.-C., Legant, W.R., Wang, K., Shao, L., Milkie, D.E., Davidson, M.W. et al. (2014) Lattice light-sheet microscopy: imaging molecules to embryos at high spatiotemporal resolution. Science 346, 1257998 10.1126/science.1257998 25342811 PMC4336192

[33] Fritzsche, M., Fernandes, R.A., Chang, V.T., Colin-York, H., Clausen, M.P., Felce, J.H. et al. (2017) Cytoskeletal actin dynamics shape a ramifying actin network underpinning immunological synapse formation. Sci. Adv. 3, e1603032 10.1126/sciadv.1603032 28691087 PMC5479650

[34] Ritter, A.T., Asano, Y., Stinchcombe, J.C., Dieckmann, N.M.G., Chen, B.-C., Gawden-Bone, C. et al. (2015) Actin depletion initiates events leading to granule secretion at the immunological synapse. Immunity 42, 864–876 10.1016/j.immuni.2015.04.013 25992860 PMC4448150

[35] Liu, T.-L., Upadhyayula, S., Milkie, D.E., Singh, V., Wang, K., Swinburne, I.A. et al. (2018) Observing the cell in its native state: imaging subcellular dynamics in multicellular organisms. Science 360, eaaq1392 10.1126/science.aaq1392 29674564 PMC6040645

[36] Yang, B., Chen, X., Wang, Y., Feng, S., Pessino, V., Stuurman, N. et al. (2019) Epi-illumination SPIM for volumetric imaging with high spatial-temporal resolution. Nat. Methods 16, 501–504 10.1038/s41592-019-0401-3 31061492 PMC6557432

[37] Sapoznik, E., Chang, B.-J., Huh, J., Ju, R.J., Azarova, E.V., Pohlkamp, T. et al. (2020) A versatile oblique plane microscope for large-scale and high-resolution imaging of subcellular dynamics. eLife 9, e57681 10.7554/eLife.57681 33179596 PMC7707824

[38] Rust, M.J., Bates, M. and Zhuang, X. (2006) Sub-diffraction-limit imaging by stochastic optical reconstruction microscopy (STORM). Nat. Methods 3, 793–795 10.1038/nmeth929 16896339 PMC2700296

[39] Heilemann, M., van de Linde, S., Schuttpelz, M., Kasper, R., Seefeldt, B., Mukherjee, A. et al. (2008) Subdiffraction-resolution fluorescence imaging with conventional fluorescent probes. Angew. Chem. Int. Ed. Engl. 47, 6172–6176 10.1002/anie.200802376 18646237

[40] Betzig, E., Patterson, G.H., Sougrat, R., Lindwasser, O.W., Olenych, S., Bonifacino, J.S. et al. (2006) Imaging intracellular fluorescent proteins at nanometer resolution. Science 313, 1642–1645 10.1126/science.1127344 16902090

[41] Hess, S.T., Girirajan, T.P. and Mason, M.D. (2006) Ultra-high resolution imaging by fluorescence photoactivation localization microscopy. Biophys. J. 91, 4258–4272 10.1529/biophysj.106.091116 16980368 PMC1635685

[42] Sharonov, A. and Hochstrasser, R.M. (2006) Wide-field subdiffraction imaging by accumulated binding of diffusing probes. Proc. Natl Acad. Sci. U.S.A. 103, 18911–18916 10.1073/pnas.0609643104 17142314 PMC1748151

[43] Fölling, J., Bossi, M., Bock, H., Medda, R., Wurm, C.A., Hein, B. et al. (2008) Fluorescence nanoscopy by ground-state depletion and single-molecule return. Nat. Methods 5, 943–945 10.1038/nmeth.1257 18794861

[44] Baumgart, F., Arnold, A.M., Rossboth, B.K., Brameshuber, M. and Schütz, G.J. (2018) What we talk about when we talk about nanoclusters. Methods Appl. Fluoresc. 7, 013001 10.1088/2050-6120/aaed0f 30412469

[45] Thompson, R.E., Larson, D.R. and Webb, W.W. (2002) Precise nanometer localization analysis for individual fluorescent probes. Biophys. J. 82, 2775–2783 10.1016/S0006-3495(02)75618-X 11964263 PMC1302065

[46] Mortensen, K.I., Churchman, L.S., Spudich, J.A. and Flyvbjerg, H. (2010) Optimized localization analysis for single-molecule tracking and super-resolution microscopy. Nat. Methods 7, 377–381 10.1038/nmeth.1447 20364147 PMC3127582

[47] Lillemeier, B.F., Mortelmaier, M.A., Forstner, M.B., Huppa, J.B., Groves, J.T. and Davis, M.M. (2009) TCR and Lat are expressed on separate protein islands on T cell membranes and concatenate during activation. Nat. Immunol. 11, 90–96 10.1038/ni.1832 20010844 PMC3273422

[48] Rossboth, B., Arnold, A.M., Ta, H., Platzer, R., Kellner, F., Huppa, J.B. et al. (2018) TCRs are randomly distributed on the plasma membrane of resting antigen-experienced T cells. Nat. Immunol. 19, 821–827 10.1038/s41590-018-0162-7 30013143 PMC6071872

[49] Velas, L., Brameshuber, M., Huppa, J.B., Kurz, E., Dustin, M.L., Zelger, P. et al. (2021) Three-Dimensional single molecule localization microscopy reveals the topography of the immunological synapse at isotropic precision below 15 nm. Nano Lett. 21, 9247–9255 10.1021/acs.nanolett.1c03160 34709845 PMC8587899

[50] Williamson, D.J., Owen, D.M., Rossy, J., Magenau, A., Wehrmann, M., Gooding, J.J. et al. (2011) Pre-existing clusters of the adaptor Lat do not participate in early T cell signaling events. Nat. Immunol. 12, 655–662 10.1038/ni.2049 21642986

[51] Rossy, J., Williamson, D.J. and Gaus, K. (2012) How does the kinase Lck phosphorylate the T cell receptor? Spatial organization as a regulatory mechanism. Front. Immunol. 3, 167 10.3389/fimmu.2012.00167 22723799 PMC3377954

[52] Baumgart, F., Arnold, A.M., Leskovar, K., Staszek, K., Folser, M., Weghuber, J. et al. (2016) Varying label density allows artifact-free analysis of membrane-protein nanoclusters. Nat. Methods 13, 661–664 10.1038/nmeth.3897 27295310 PMC6404959

[53] Sherman, E., Barr, V., Manley, S., Patterson, G., Balagopalan, L., Akpan, I. et al. (2011) Functional nanoscale organization of signaling molecules downstream of the T cell antigen receptor. Immunity 35, 705–720 10.1016/j.immuni.2011.10.004 22055681 PMC3225724

[54] Sherman, E., Barr, V. and Samelson, L.E. (2013) Super-resolution characterization of TCR-dependent signaling clusters. Immunol. Rev. 251, 21–35 10.1111/imr.12010 23278738 PMC3539238

[55] Gomes de Castro, M.A., Wildhagen, H., Sograte-Idrissi, S., Hitzing, C., Binder, M., Trepel, M. et al. (2019) Differential organization of tonic and chronic B cell antigen receptors in the plasma membrane. Nat. Commun. 10, 820 10.1038/s41467-019-08677-1 30778055 PMC6379438

[56] Stone, M.B., Shelby, S.A., Nunez, M.F., Wisser, K. and Veatch, S.L. (2017) Protein sorting by lipid phase-like domains supports emergent signaling function in B lymphocyte plasma membranes. eLife 6, e19891 10.7554/eLife.19891 28145867 PMC5373823

[57] Pageon, S.V., Cordoba, S.-P., Owen, D.M., Rothery, S.M., Oszmiana, A. and Davis, D.M. (2013) Superresolution microscopy reveals nanometer-scale reorganization of inhibitory natural killer cell receptors upon activation of NKG2D. Sci. Signal. 6, ra62 10.1126/scisignal.2003947 23882121

[58] Oheim, M., Salomon, A. and Brunstein, M. (2020) Supercritical angle fluorescence microscopy and spectroscopy. Biophys. J. 118, 2339–2348 10.1016/j.bpj.2020.03.029 32348720 PMC7231923

[59] Bourg, N., Mayet, C., Dupuis, G., Barroca, T., Bon, P., Lécart, S. et al. (2015) Direct optical nanoscopy with axially localized detection. Nat. Photon 9, 587–593 10.1038/nphoton.2015.132

[60] Brameshuber, M. and Schütz, G.J. (2008) How the sum of its parts gets greater than the whole. Nat. Methods 5, 133–134 10.1038/nmeth0208-133 18235433

[61] Tanaka, K.A.K., Suzuki, K.G.N., Shirai, Y.M., Shibutani, S.T., Miyahara, M.S.H., Tsuboi, H. et al. (2010) Membrane molecules mobile even after chemical fixation. Nat. Methods 7, 865–866 10.1038/nmeth.f.314 20881966

[62] Hoffman, D.P., Shtengel, G., Xu, C.S., Campbell, K.R., Freeman, M., Wang, L. et al. (2020) Correlative three-dimensional super-resolution and block-face electron microscopy of whole vitreously frozen cells. Science 367, eaaz5357 10.1126/science.aaz5357 31949053 PMC7339343

[63] Klar, T.A., Jakobs, S., Dyba, M., Egner, A. and Hell, S.W. (2000) Fluorescence microscopy with diffraction resolution barrier broken by stimulated emission. Proc. Natl Acad. Sci. U.S.A. 97, 8206–8210 10.1073/pnas.97.15.8206 10899992 PMC26924

[64] Bretschneider, S., Eggeling, C. and Hell, S.W. (2007) Breaking the diffraction barrier in fluorescence microscopy by optical shelving. Phys. Rev. Lett. 98, 218103 10.1103/PhysRevLett.98.218103 17677813

[65] Wieser, S. and Schütz, G.J. (2008) Tracking single molecules in the live cell plasma membrane-do's and don't’s. Methods 46, 131–140 10.1016/j.ymeth.2008.06.010 18634880

[66] Umemura, Y.M., Vrljic, M., Nishimura, S.Y., Fujiwara, T.K., Suzuki, K.G.N. and Kusumi, A. (2008) Both MHC class II and its GPI-anchored form undergo hop diffusion as observed by single-molecule tracking. Biophys. J. 95, 435–450 10.1529/biophysj.107.123018 18339737 PMC2426619

[67] Huppa, J.B., Axmann, M., Mörtelmaier, M.A., Lillemeier, B.F., Newell, E.W., Brameshuber, M. et al. (2010) TCR-peptide-MHC interactions in situ show accelerated kinetics and increased affinity. Nature 463, 963–967 10.1038/nature08746 20164930 PMC3273423

[68] O'Donoghue, G.P., Pielak, R.M., Smoligovets, A.A., Lin, J.J. and Groves, J.T. (2013) Direct single molecule measurement of TCR triggering by agonist pMHC in living primary T cells. eLife 2, e00778 10.7554/eLife.00778 23840928 PMC3701909

[69] Katz, Z.B., Novotná, L., Blount, A. and Lillemeier, B.F. (2017) A cycle of Zap70 kinase activation and release from the TCR amplifies and disperses antigenic stimuli. Nat. Immunol. 18, 86–95 10.1038/ni.3631 27869819 PMC5490839

[70] Brameshuber, M., Kellner, F., Rossboth, B.K., Ta, H., Alge, K., Sevcsik, E. et al. (2018) Monomeric TCRs drive T cell antigen recognition. Nat. Immunol. 19, 487–496 10.1038/s41590-018-0092-4 29662172 PMC7612939

[71] Göhring, J., Kellner, F., Schrangl, L., Platzer, R., Klotzsch, E., Stockinger, H. et al. (2021) Temporal analysis of T-cell receptor-imposed forces via quantitative single molecule FRET measurements. Nat. Commun. 12, 2502 10.1038/s41467-021-22775-z 33947864 PMC8096839

[72] Huang, J., Zarnitsyna, V.I., Liu, B., Edwards, L.J., Jiang, N., Evavold, B.D. et al. (2010) The kinetics of two-dimensional TCR and pMHC interactions determine T-cell responsiveness. Nature 464, 932–936 10.1038/nature08944 20357766 PMC2925443

[73] Sims, T.N., Soos, T.J., Xenias, H.S., Dubin-Thaler, B., Hofman, J.M., Waite, J.C. et al. (2007) Opposing effects of PKCtheta and WASp on symmetry breaking and relocation of the immunological synapse. Cell 129, 773–785 10.1016/j.cell.2007.03.037 17512410

[74] Klotzsch, E., Stiegler, J., Ben-Ishay, E. and Gaus, K. (2015) Do mechanical forces contribute to nanoscale membrane organisation in T cells? Biochim. Biophys. Acta 1853, 822–829 10.1016/j.bbamcr.2014.10.025 25447546

[75] Klotzsch, E. and Schütz, G.J. (2013) Improved ligand discrimination by force-induced unbinding of the T cell receptor from peptide-MHC. Biophys. J. 104, 1670–1675 10.1016/j.bpj.2013.03.023 23601314 PMC3628311

[76] Husson, J., Chemin, K., Bohineust, A., Hivroz, C. and Henry, N. (2011) Force generation upon T cell receptor engagement. PLoS ONE 6, e19680 10.1371/journal.pone.0019680 21572959 PMC3091878

[77] Hu, K.H. and Butte, M.J. (2016) T cell activation requires force generation. J. Cell Biol. 213, 535–542 10.1083/jcb.201511053 27241914 PMC4896056

[78] Liu, B., Chen, W., Evavold, B.D. and Zhu, C. (2014) Accumulation of dynamic catch bonds between TCR and agonist peptide-MHC triggers T cell signaling. Cell 157, 357–368 10.1016/j.cell.2014.02.053 24725404 PMC4123688

[79] Limozin, L., Bridge, M., Bongrand, P., Dushek, O., van der Merwe, P.A. and Robert, P. (2019) TCR-pMHC kinetics under force in a cell-free system show no intrinsic catch bond, but a minimal encounter duration before binding. Proc. Natl Acad. Sci. U.S.A. 116, 16943–16948 10.1073/pnas.1902141116 31315981 PMC6708305

[80] Gourier, C., Jegou, A., Husson, J. and Pincet, F. (2008) A nanospring named erythrocyte. The biomembrane force probe. Cell. Mol. Bioeng. 1, 263 10.1007/s12195-008-0030-x

[81] Kaizuka, Y., Douglass, A.D., Varma, R., Dustin, M.L. and Vale, R.D. (2007) Mechanisms for segregating T cell receptor and adhesion molecules during immunological synapse formation in jurkat T cells. Proc. Natl Acad. Sci. U.S.A. 104, 20296–20301 10.1073/pnas.0710258105 18077330 PMC2154425

[82] Feng, Y., Brazin, K.N., Kobayashi, E., Mallis, R.J., Reinherz, E.L. and Lang, M.J. (2017) Mechanosensing drives acuity of αβ T-cell recognition. Proc. Natl Acad. Sci. U.S.A. 114, E8204–E8213 10.1073/pnas.1703559114 28811364 PMC5625899

[83] Li, Y.-C., Chen, B.-M., Wu, P.-C., Cheng, T.-L., Kao, L.-S., Tao, M.-H. et al. (2010) Cutting edge: mechanical forces acting on T cells immobilized via the TCR complex can trigger TCR signaling. J. Immunol. 184, 5959–5963 10.4049/jimmunol.0900775 20435924

[84] Judokusumo, E., Tabdanov, E., Kumari, S., Dustin, M.L. and Kam, L.C. (2012) Mechanosensing in T lymphocyte activation. Biophys. J. 102, L5–L7 10.1016/j.bpj.2011.12.011 22339876 PMC3260692

[85] Bashour, K.T., Gondarenko, A., Chen, H., Shen, K., Liu, X., Huse, M. et al. (2014) CD28 and CD3 have complementary roles in T-cell traction forces. Proc. Natl Acad. Sci. U.S.A. 111, 2241–2246 10.1073/pnas.1315606111 24469820 PMC3926067

[86] Colin-York, H., Javanmardi, Y., Skamrahl, M., Kumari, S., Chang, V.T., Khuon, S. et al. (2019) Cytoskeletal control of antigen-Dependent T cell activation. Cell Rep. 26, 3369–3379.e5 10.1016/j.celrep.2019.02.074 30893608 PMC6426652

[87] Wang, J., Lin, F., Wan, Z., Sun, X., Lu, Y., Huang, J. et al. (2018) Profiling the origin, dynamics, and function of traction force in B cell activation. Sci. Signal. 11, eaai9192 10.1126/scisignal.aai9192 30087179

[88] Barbieri, L., Colin-York, H., Korobchevskaya, K., Li, D., Wolfson, D.L., Karedla, N. et al. (2021) Two-dimensional TIRF-SIM-traction force microscopy (2D TIRF-SIM-TFM). Nat. Commun. 12, 2169 10.1038/s41467-021-22377-9 33846317 PMC8041833

[89] Aramesh, M., Mergenthal, S., Issler, M., Plochberger, B., Weber, F., Qin, X.-H. et al. (2021) Functionalized bead assay to measure three-dimensional traction forces during T-cell activation. Nano Lett. 21, 507–514 10.1021/acs.nanolett.0c03964 33305952

[90] Liu, Y., Blanchfield, L., Ma, V.P.-Y., Andargachew, R., Galior, K., Liu, Z. et al. (2016) DNA-based nanoparticle tension sensors reveal that T-cell receptors transmit defined pN forces to their antigens for enhanced fidelity. Proc. Natl Acad. Sci. U.S.A. 113, 5610–5615 10.1073/pnas.1600163113 27140637 PMC4878516

[91] Ma, V.P.-Y., Liu, Y., Blanchfield, L., Su, H., Evavold, B.D. and Salaita, K. (2016) Ratiometric tension probes for mapping receptor forces and clustering at intermembrane junctions. Nano Lett. 16, 4552–4559 10.1021/acs.nanolett.6b01817 27192323 PMC6061938

[92] Cocco, S., Monasson, R. and Marko, J.F. (2001) Force and kinetic barriers to unzipping of the DNA double helix. Proc. Natl Acad. Sci. U.S.A. 98, 8608–8613 10.1073/pnas.151257598 11447279 PMC37483

[93] Mosayebi, M., Louis, A.A., Doye, J.P.K. and Ouldridge, T.E. (2015) Force-induced rupture of a DNA duplex: from fundamentals to force sensors. ACS Nano 9, 11993–12003 10.1021/acsnano.5b04726 26575598

[94] Grashoff, C., Hoffman, B.D., Brenner, M.D., Zhou, R., Parsons, M., Yang, M.T. et al. (2010) Measuring mechanical tension across vinculin reveals regulation of focal adhesion dynamics. Nature 466, 263 10.1038/nature09198 20613844 PMC2901888

[95] Toepfner, N., Herold, C., Otto, O., Rosendahl, P., Jacobi, A., Krater, M. et al. (2018) Detection of human disease conditions by single-cell morpho-rheological phenotyping of blood. eLife 7, e29213 10.7554/eLife.29213 29331015 PMC5790376

[96] Kubánková, M., Hohberger, B., Hoffmanns, J., Fürst, J., Herrmann, M., Guck, J. et al. (2021) Physical phenotype of blood cells is altered in COVID-19. Biophys. J. 120, 2838–2847 10.1016/j.bpj.2021.05.025 34087216 PMC8169220

[97] Sezgin, E., Schneider, F., Zilles, V., Urbancic, I., Garcia, E., Waithe, D. et al. (2017) Polarity-sensitive probes for superresolution stimulated emission depletion microscopy. Biophys. J. 113, 1321–1330 10.1016/j.bpj.2017.06.050 28734477 PMC5607142

[98] Kuimova, M.K. (2012) Mapping viscosity in cells using molecular rotors. Phys. Chem. Chem. Phys. 14, 12671–12686 10.1039/c2cp41674c 22806312

[99] Colom, A., Derivery, E., Soleimanpour, S., Tomba, C., Dal Molin, M., Sakai, N. et al. (2018) A fluorescent membrane tension probe. Nat. Chem. 10, 1118–1125 10.1038/s41557-018-0127-3 30150727 PMC6197433

[100] Jung, P., Zhou, X., Iden, S., Bischoff, M. and Qu, B. (2021) T cell stiffness is enhanced upon formation of immunological synapse. eLife 10, e66643 10.7554/eLife.66643 34313220 PMC8360652

[101] Rosendahl, P., Plak, K., Jacobi, A., Kraeter, M., Toepfner, N., Otto, O. et al. (2018) Real-time fluorescence and deformability cytometry. Nat. Methods 15, 355–358 10.1038/nmeth.4639 29608556

[102] Nyberg, K.D., Hu, K.H., Kleinman, S.H., Khismatullin, D.B., Butte, M.J. and Rowat, A.C. (2017) Quantitative deformability cytometry: rapid, calibrated measurements of cell mechanical properties. Biophys. J. 113, 1574–1584 10.1016/j.bpj.2017.06.073 28978449 PMC5627151

[103] Lühr, J.J., Alex, N., Amon, L., Kräter, M., Kubánková, M., Sezgin, E. et al. (2020) Maturation of monocyte-Derived DCs leads to increased cellular stiffness, higher membrane fluidity, and changed lipid composition. Front. Immunol. 11, 590121 10.3389/fimmu.2020.590121 33329576 PMC7728921

[104] Matias, M.I., Yong, C.S., Foroushani, A., Goldsmith, C., Mongellaz, C., Sezgin, E. et al. (2021) Regulatory T cell differentiation is controlled by αKG-induced alterations in mitochondrial metabolism and lipid homeostasis. Cell Rep. **37**, 109911 10.1016/j.celrep.2021.109911 PMC10167917 34731632

[105] Hu, Z.-Q., Xue, H., Long, J.-H., Wang, Y., Jia, Y., Qiu, W. et al. (2016) Biophysical properties and motility of human mature dendritic cells deteriorated by vascular endothelial growth factor through cytoskeleton remodeling. Int. J. Mol. Sci. 17, 1756 10.3390/ijms17111756 PMC5133777 27809226

[106] Zhang, T., Hu, W. and Chen, W. (2021) Plasma membrane integrates biophysical and biochemical regulation to trigger immune receptor functions. Front. Immunol. 12, 53 10.3389/fimmu.2021.613185 PMC7933204 33679752

[107] Bufi, N., Saitakis, M., Dogniaux, S., Buschinger, O., Bohineust, A., Richert, A. et al. (2015) Human primary immune cells exhibit distinct mechanical properties that Are modified by inflammation. Biophys. J. 108, 2181–2190 10.1016/j.bpj.2015.03.047 25954876 PMC4423053

[108] Tello-Lafoz, M., Srpan, K., Sanchez, E.E., Hu, J., Remsik, J., Romin, Y. et al. (2021) Cytotoxic lymphocytes target characteristic biophysical vulnerabilities in cancer. Immunity 54, 1037–1054.e7 10.1016/j.immuni.2021.02.020 33756102 PMC8119359

[109] Jin, W., Tamzalit, F., Chaudhuri, P.K., Black, C.T., Huse, M. and Kam, L.C. (2019) T cell activation and immune synapse organization respond to the microscale mechanics of structured surfaces. Proc. Natl Acad. Sci. U.S.A. 116, 19835–19840 10.1073/pnas.1906986116 31527238 PMC6778209

[110] Mennens, S.F.B., Bolomini-Vittori, M., Weiden, J., Joosten, B., Cambi, A. and van den Dries, K. (2017) Substrate stiffness influences phenotype and function of human antigen-presenting dendritic cells. Sci. Rep. 7, 17511 10.1038/s41598-017-17787-z 29235514 PMC5727489

[111] Rentero, C., Zech, T., Quinn, C.M., Engelhardt, K., Williamson, D., Grewal, T. et al. (2008) Functional implications of plasma membrane condensation for T cell activation. PLoS ONE 3, e2262 10.1371/journal.pone.0002262 18509459 PMC2384009

[112] Hivroz, C. and Saitakis, M. (2016) Biophysical aspects of T lymphocyte activation at the immune synapse. Front. Immunol. 7, 46 10.3389/fimmu.2016.00046 26913033 PMC4753286

[113] Owen, D.M., Oddos, S., Kumar, S., Davis, D.M., Neil, M.A.A., French, P.M.W. et al. (2010) High plasma membrane lipid order imaged at the immunological synapse periphery in live T cells. Mol. Membr. Biol. 27, 178–189 10.3109/09687688.2010.495353 20540668 PMC3870023

[114] Li, Y. and Orange, J.S. (2021) Degranulation enhances presynaptic membrane packing, which protects NK cells from perforin-mediated autolysis. PLoS Biol. 19, e3001328 10.1371/journal.pbio.3001328 34343168 PMC8330931

[115] Rudd-Schmidt, J.A., Hodel, A.W., Noori, T., Lopez, J.A., Cho, H.-J., Verschoor, S. et al. (2019) Lipid order and charge protect killer T cells from accidental death. Nat. Commun. 10, 5396 10.1038/s41467-019-13385-x 31776337 PMC6881447

[116] Felce, J., Sezgin, E., Wane, M., Brouwer, H., Dustin, M.L., Eggeling, C. et al. (2018) CD45 exclusion and cross-linking based receptor signaling together broaden FcεRI reactivity. Sci. Signal. 11, eaat0756 10.1126/scisignal.aat0756 30563863 PMC7612966

[117] Bakalar, M.H., Joffe, A.M., Schmid, E.M., Son, S., Podolski, M. and Fletcher, D.A. (2018) Size-dependent segregation controls macrophage phagocytosis of antibody-opsonized targets. Cell 174, 131–142.e13 10.1016/j.cell.2018.05.059 29958103 PMC6067926

[118] Urbančič, I., Schiffelers, L., Jenkins, E., Gong, W., Santos, A.M., Schneider, F. et al. (2021) Aggregation and mobility of membrane proteins interplay with local lipid order in the plasma membrane of T cells. FEBS Lett. 595, 2127–2146 10.1002/1873-3468.14153 34160065

[119] Froimchuk, E., Oakes, R.S., Kapnick, S.M., Yanes, A.A. and Jewell, C.M. (2021) Biophysical properties of self-assembled immune signals impact signal processing and the nature of regulatory immune function. Nano Lett. 21, 3762–3771 10.1021/acs.nanolett.0c05118 33881872 PMC8119350

[120] Hu, Y.S., Cang, H. and Lillemeier, B.F. (2016) Superresolution imaging reveals nanometer- and micrometer-scale spatial distributions of T-cell receptors in lymph nodes. Proc. Natl Acad. Sci. U.S.A. 113, 7201–7206 10.1073/pnas.1512331113 27303041 PMC4932922

[121] Platzer, R., Rossboth, B.K., Schneider, M.C., Sevcsik, E., Baumgart, F., Stockinger, H. et al. (2020) Unscrambling fluorophore blinking for comprehensive cluster detection via photoactivated localization microscopy. Nat. Commun. 11, 4993 10.1038/s41467-020-18726-9 33020470 PMC7536177

[122] Spahn, C., Herrmannsdorfer, F., Kuner, T. and Heilemann, M. (2016) Temporal accumulation analysis provides simplified artifact-free analysis of membrane-protein nanoclusters. Nat. Methods 13, 963–964 10.1038/nmeth.4065 27898062

[123] Arnold, A.M., Schneider, M.C., Hüsson, C., Sablatnig, R., Brameshuber, M., Baumgart, F. et al. (2020) Verifying molecular clusters by 2-color localization microscopy and significance testing. Sci. Rep. 10, 4230 10.1038/s41598-020-60976-6 32144344 PMC7060173

[124] Li, D., Colin-York, H., Barbieri, L., Javanmardi, Y., Guo, Y., Korobchevskaya, K. et al. (2021) Astigmatic traction force microscopy (aTFM). Nat. Commun. 12, 2168 10.1038/s41467-021-22376-w 33846322 PMC8042066

[125] Yi, J., Manna, A., Barr, V.A., Hong, J., Neuman, K.C. and Samelson, L.E. (2016) madSTORM: a superresolution technique for large-scale multiplexing at single-molecule accuracy. Mol. Biol. Cell 27, 3591–3600 10.1091/mbc.e16-05-0330 27708141 PMC5221591

[126] Bodén, A., Pennacchietti, F., Coceano, G., Damenti, M., Ratz, M. and Testa, I. (2021) Volumetric live cell imaging with three-dimensional parallelized RESOLFT microscopy. Nat. Biotechnol. 39, 609–618 10.1038/s41587-020-00779-2 33432197

[127] Hoyer, P., de Medeiros, G., Balázs, B., Norlin, N., Besir, C., Hanne, J. et al. (2016) Breaking the diffraction limit of light-sheet fluorescence microscopy by RESOLFT. Proc. Natl Acad. Sci. U.S.A. 113, 3442–3446 10.1073/pnas.1522292113 26984498 PMC4822606

[128] Cao, B., Coelho, S., Li, J., Wang, G. and Pertsinidis, A. (2021) Volumetric interferometric lattice light-sheet imaging. Nat. Biotechnol. 39, 1385–1393 10.1038/s41587-021-01042-y 34635835 PMC8595582

[129] Nehme, E., Freedman, D., Gordon, R., Ferdman, B., Weiss, L.E., Alalouf, O. et al. (2020) DeepSTORM3D: dense 3D localization microscopy and PSF design by deep learning. Nat. Methods 17, 734–740 10.1038/s41592-020-0853-5 32541853 PMC7610486

[130] Speiser, A., Müller, L.-R., Hoess, P., Matti, U., Obara, C.J., Legant, W.R. et al. (2021) Deep learning enables fast and dense single-molecule localization with high accuracy. Nat. Methods 18, 1082–1090 10.1038/s41592-021-01236-x 34480155 PMC7611669

[131] Diekmann, R., Kahnwald, M., Schoenit, A., Deschamps, J., Matti, U. and Ries, J. (2020) Optimizing imaging speed and excitation intensity for single-molecule localization microscopy. Nat. Methods 17, 909–912 10.1038/s41592-020-0918-5 32807954 PMC7610360

[132] Chung, K.K., Zhang, Z., Kidd, P., Zhang, Y., Williams, N.D., Rollins, B. et al. (2020) Fluorogenic probe for fast 3D whole-cell DNA-PAINT. bioRxiv 10.1101/2020.04.29.066886

[133] Balzarotti, F., Eilers, Y., Gwosch, K.C., Gynna, A.H., Westphal, V., Stefani, F.D. et al. (2017) Nanometer resolution imaging and tracking of fluorescent molecules with minimal photon fluxes. Science 355, 606–612 10.1126/science.aak9913 28008086

[134] Bingen, P., Reuss, M., Engelhardt, J. and Hell, S.W. (2011) Parallelized STED fluorescence nanoscopy. Opt. Express 19, 23716–23726 10.1364/OE.19.023716 22109398

[135] Collins, J.T., Knapper, J., Stirling, J., Mduda, J., Mkindi, C., Mayagaya, V. et al. (2020) Robotic microscopy for everyone: the openFlexure microscope. Biomed. Opt. Express 11, 2447–2460 10.1364/BOE.385729 32499936 PMC7249832

[136] Diederich, B., Lachmann, R., Carlstedt, S., Marsikova, B., Wang, H., Uwurukundo, X. et al. (2020) A versatile and customizable low-cost 3D-printed open standard for microscopic imaging. Nat. Commun. 11, 5979 10.1038/s41467-020-19447-9 33239615 PMC7688980

[137] von Chamier, L., Laine, R.F., Jukkala, J., Spahn, C., Krentzel, D., Nehme, E. et al. (2021) Democratising deep learning for microscopy with ZeroCostDL4Mic. Nat. Commun. 12, 2276 10.1038/s41467-021-22518-0 33859193 PMC8050272

[138] Halpern, A.R., Lee, M.Y., Howard, M.D., Woodworth, M.A., Nicovich, P.R. and Vaughan, J.C. (2021) Versatile, do-it-yourself, low-cost spinning disk confocal microscope. Biomed. Opt. Express **13**, 1102–1120 10.1364/BOE.442087 PMC8884209 35284165

[139] Li, H., Krishnamurthy, D., Li, E., Vyas, P., Akireddy, N., Chai, C. et al. (2020) Squid: simplifying quantitative imaging platform development and deployment. bioRxiv 10.1101/2020.12.28.424613

[140] Gregor, I., Butkevich, E., Enderlein, J. and Mojiri, S. (2021) Instant three-color multiplane fluorescence microscopy. Biophys. Rep. 1, 100001 10.1016/j.bpr.2021.100001 PMC9680778 36425311

[141] Schröder, D., Deschamps, J., Dasgupta, A., Matti, U. and Ries, J. (2020) Cost-efficient open source laser engine for microscopy. Biomed. Opt. Express 11, 609–623 10.1364/BOE.380815 32206389 PMC7041445

[142] Stanly, T.A., Fritzsche, M., Banerji, S., Garcia, E., Bernardino de la Serna, J., Jackson, D.G. et al. (2016) Critical importance of appropriate fixation conditions for faithful imaging of receptor microclusters. Biol. Open 5, 1343–1350 10.1242/bio.019943 27464671 PMC5051640

[143] Büttner, M., Lagerholm, C.B., Waithe, D., Galiani, S., Schliebs, W., Erdmann, R. et al. (2021) Challenges of using expansion microscopy for super-resolved imaging of cellular organelles. Chembiochem 22, 686–693 10.1002/cbic.202000571 33049107 PMC7894168

[144] Chang, Y.-W., Chen, S., Tocheva, E.I., Treuner-Lange, A., Löbach, S., Søgaard-Andersen, L. et al. (2014) Correlated cryogenic photoactivated localization microscopy and cryo-electron tomography. Nat. Methods 11, 737–739 10.1038/nmeth.2961 24813625 PMC4081473

[145] Pasqual, G., Chudnovskiy, A., Tas, J.M.J., Agudelo, M., Schweitzer, L.D., Cui, A. et al. (2018) Monitoring T cell-dendritic cell interactions in vivo by intercellular enzymatic labelling. Nature 553, 496–500 10.1038/nature25442 29342141 PMC5853129

[146] Jenkins, E., Santos, A.M., O'Brien-Ball, C., Felce, J.H., Wilcock, M.J., Hatherley, D. et al. (2018) Reconstitution of immune cell interactions in free-standing membranes. J. Cell Sci. 132, jcs219709 10.1242/jcs.219709 30209137 PMC6398472

